# The Predictive Value of the aCL and Anti-β2GPI at the Time of Acute Deep Vein Thrombosis—A Two-Year Prospective Study

**DOI:** 10.3390/biomedicines9080901

**Published:** 2021-07-27

**Authors:** Katja Perdan-Pirkmajer, Polona Žigon, Anja Boc, Eva Podovšovnik, Saša Čučnik, Alenka Mavri, Žiga Rotar, Aleš Ambrožič

**Affiliations:** 1 Department of Rheumatology, University Medical Centre Ljubljana, SI-1000 Ljubjana, Slovenia; katja.perdan@mf.uni-lj.si (K.P.-P.); polona.zigon@guest.arnes.si (P.Ž.); sasa.cucnik@guest.arnes.si (S.Č.); ziga.rotar@kclj.si (Ž.R.); 2 Division for Internal Medicine, Faculty of Medicine, University of Ljubljana, SI-1000 Ljubljana, Slovenia; alenka.mavri@kclj.si; 3 Faculty of Mathematics, Natural Sciences and Information Technologies, University of Primorska, SI-6000 Koper, Slovenia; 4 Faculty of Medicine, Institute of Anatomy, University of Ljubljana, SI-1000 Ljubljana, Slovenia; anja.boc@mf.uni-lj.si; 5 Department of Vascular Diseases, University Medical Centre Ljubljana, SI-1000 Ljubljana, Slovenia; 6 Faculty of Tourism Studies, University of Primorska, SI-6000 Portorož, Slovenia; eva.podovsovnik@gmail.com; 7 Faculty of Pharmacy, University of Ljubljana, SI-1000 Ljubljana, Slovenia

**Keywords:** thrombosis, antiphospholipid syndrome, antiphospholipid antibodies, prediction

## Abstract

Antiphospholipid syndrome (APS) is an important cause of deep vein thrombosis (DVT). According to current APS classification criteria, APS cannot be confirmed until 24 weeks after DVT. This time frame results in frequent discontinuation of anticoagulant treatment before APS is diagnosed. Therefore, the aim of our study was to evaluate the potential predictive value of anticardiolipin (aCL) and anti-β2glycoprotein I (anti-β2GPI) before discontinuation of anticoagulation therapy. Patients with newly diagnosed DVT were included into a 24-month prospective study. All patients received anticoagulant therapy. aCL and anti-β2GPI were determined at inclusion and every four weeks for the first 24 weeks and then one and two years after inclusion. APS was confirmed in 24/221 (10.9%) patients. At the time of acute DVT 20/24 (83.3%), APS patients had positive aCL and/or anti-β2GPI. Two patients had low aCL levels and two were negative at the time of acute DVT but later met APS criteria due to lupus anticoagulant (LA). Our data indicate that negative aCL and/or anti-β2GPI at the time of acute DVT make further aPL testing unnecessary; however, LA should be determined after discontinuation of anticoagulant therapy. Positive aCL and/or anti-β2GPI at the time of acute DVT have a strong positive predictive value for APS and may support therapeutic decisions.

## 1. Introduction

Antiphospholipid syndrome (APS) is a systemic autoimmune disease characterized by thrombotic events and/or pregnancy morbidity and persistent presence of antiphospholipid antibodies (aPL) [1]. It is estimated that 1 to 5% of healthy individuals have aPL, while the incidence of APS is approximately 5 cases per 100,000 persons per year, and the prevalence is approximately 40–50 cases per 100,000 persons [2]. Common APS clinical presentations include venous thromboembolism, stroke, recurrent early miscarriages and late pregnancy losses/complications [3]. Characteristic laboratory abnormalities in APS include persistently elevated levels of antibodies directed against membrane anionic phospholipids (e.g., anticardiolipin antibody (aCL)) or their associated plasma proteins, predominantly beta-2 glycoprotein I (β2GPI), or evidence of a circulating lupus anticoagulant (LA).

Currently, there are no diagnostic APS criteria, however, according to the revised classification criteria proposed as an international consensus statement at a workshop in Sydney in 2006, definite APS is present if at least one of the clinical criteria and one of the laboratory criteria are met [3]. Regarding the laboratory APS criteria, LA, aCL of IgG and/or IgM isotype (titres >40 IgG phospholipid (GPL) units or >40 IgM phospholipid (MPL) units, or >the 99th percentile, measured by a standardized ELISA) or anti-β2GPI of IgG and/or IgM isotype (titre > the 99th percentile, measured by a standardized ELISA) count [3]. Furthermore, some patients with clinical manifestations highly suggestive of APS are negative for criteria biomarkers and anti-phosphatidylserine/prothrombin (aPS/PT) antibodies have been found positive in many seronegative patients [4,5]. While anticoagulant therapy affects LA determination, it does not affect the determination of aPL by ELISA. In addition, the persistence of the autoantibodies is important, as aPL may be transiently elevated by infection, malignancy or certain medications [6,7]; therefore, repeated testing at an interval of 12 or more weeks is required to confirm the presence of circulating aPL when a diagnosis of APS is made. Furthermore, because an acute thrombotic event may affect aPL levels [8], it was suggested that there should be an interval of at least 12 weeks between the clinical event and the first positive laboratory test. However, the proposed time intervals were based on expert opinion and further studies evaluating suggested time frames were required [3].

Deep vein thrombosis (DVT) is a major APS manifestation. In the general population, DVT and pulmonary embolism are considered diseases of aging, rising rapidly after the age of 45 years, and occurring at an annual incidence of about 1 per 1000 adults at the age of 80 years [9]. Major risk factors for thrombosis are also surgery, hospitalization, immobility, trauma, pregnancy and the puerperium, hormone use and endogenous factors such as cancer, obesity and inherited and acquired disorders of hypercoagulation [10]. Venous thrombosis is often a chronic condition, with recurrence rates estimated at 5–7% annually after the first episode [11], and the risk of recurrence seems to be greatest 6–12 months after cessation of anticoagulant therapy. This risk is even higher in APS (17% within the first year) [12]. For patients with clearly provoked DVT, current guidelines recommend anticoagulation for three months. For unprovoked first thrombotic events, a decision on whether to prolong anticoagulation therapy is often made after the first three months [13], a time frame in which definite APS diagnosis cannot yet be established. Furthermore, current DVT guidelines support the use of direct oral anticoagulants (DOACs) as the initial treatment choice. However, the prevalence of aPL following the first unprovoked thrombotic event is 9–15% [14,15], and thus it is likely that many of these patients have undiagnosed APS. Following current DVT treatment guidelines in patients with undiagnosed APS leads to an inappropriate selection of DOACs instead of vitamin K antagonists (VKA) [16,17], and cessation of anticoagulant treatment highly increases the risk for DVT recurrence. Early diagnosis of APS is therefore mandatory for appropriate selection of anticoagulant therapy and the decision about treatment cessation.

Thus, in the diagnosis and management of APS patients’, clinicians stumble upon numerous pitfalls. Recognition of a possible APS patient at the time of acute first DVT is challenging and there is a lack of data on the value of aPL determination at the time of an acute thrombotic event. The present prospective study was designed to evaluate the possible predictive value of aCL and anti-β2GPI for APS at the time of acute DVT and to evaluate the possible added value of non-criteria aPL testing (i.e., aPS/PT, IgA aCL and IgA anti-β2GPI) in the diagnostic management of DVT at the acute event. Our data suggest that determining aPL at the time of acute event can aid therapeutic decisions.

## 2. Materials and Methods

### 2.1. Setting

We conducted a 24-month single centre prospective observational study at the Department of Vascular diseases and the Department of Rheumatology, University Medical Centre Ljubljana (UMC-LJ), Ljubljana, Slovenia. UMC-LJ provides secondary level medical care for approximately 530,000 adult residents of Ljubljana region, and tertiary level medical care for approximately half of the entire Slovenian population, counting two million residents.

Adults aged ≥18 years with first episode of acute venous thromboembolism in the form of DVT were included. Patients with known or subsequently discovered malignant disease, pregnant patients and patients with recurrent deep vein thrombosis were not included in our study. Immediately after DVT confirmation, patients were treated with warfarin and subcutaneous injections of low molecular weight heparin (LMWH); injections were discontinued after international normalized ratio (INR) between 2 and 3 was achieved. Anticoagulant therapy was continued for three to six months for distal or proximal DVT, respectively. In patients with suspected APS (positive aPL during first three months) and confirmation of APS six months after DVT, anticoagulant therapy was continued. Data on possible recurrent venous thromboembolism were collected at follow-up visits. The study was approved by the National Medical Ethics Committee, Ljubljana, Slovenia (permission number 134/09/08) and all patients provided informed consent.

### 2.2. Diagnostic Work-Up

Patients were admitted to the daily hospital of Department of Vascular diseases. Thorough clinical evaluation was performed and DVT was confirmed by a compression ultrasound. Data on possible exogenous and endogenous risk factors for venous thrombosis were collected, including the screening for occult malignancy. After that, patients were referred to the Department of Rheumatology, where they underwent an extensive laboratory work-up. Laboratory investigations were performed at presentation, then every four weeks during the next 24 weeks, and finally one and two years after inclusion. Patients with at least five visits (preferably at 0 weeks, 4 weeks, 12 weeks, 24 weeks and 24 months) were later analysed. APS was confirmed according to the Sydney Criteria [3] if a patient had met the criteria for aPL 12 and 24 weeks or later after DVT.

### 2.3. Antiphospholipid Antibodies Determination

aPL, specifically aCL, anti-β2GPI and aPS/PT, were determined in the patient serum samples at the time of the acute thrombotic event and at follow-up visits. A value above the 99th percentile of the healthy control population was considered significant. Values between the 95th and 99th were considered low positive. Due to inaccurate determination of LA during anticoagulant treatment, this type of aPL was determined only after cessation of anticoagulant treatment.

In-house aCL ELISA: IgG, IgM and IgA aCL were determined according to the previously described method [18]. Briefly, medium binding microtiter plates (Corning Incorporated, Kennebunk, ME, USA) were coated with cardiolipin (Sigma-Aldrich, St. Louis, MO, USA, ZDA) and blocked with 10% fetal bovine serum (FBS, Sigma-Aldrich, St. Louis, ZDA) in phosphate-buffered saline (PBS). After washing with PBS, diluted samples in 10% FBS-PBS were applied and incubated at room temperature (RT) for 2.5 h. Alkaline phosphatase-conjugated goat anti-human IgG, IgM and IgA (ACSC, Westbury, NY, USA) and para-nitro phenyl phosphate (Sigma Chemical Company, St. Louis, MI, USA) in diethanolamine buffer (pH 9.8) were used as the detection system, and OD_405_ was measured kinetically with a spectrometer (Tecan Sunrise Remote, Groedig, Austria).

In-house anti-β2GPI ELISA was performed as previously described [19] and evaluated by the European Forum for aPL [20]. Briefly, high-binding polystyrene microtiter plates (Corning Incorporated, Kennebunk, ME, USA) coated with β2GPI (10 mg/L) in PBS were incubated for two hours at RT. Plates were then washed with PBS containing 0.05% Tween-20 (PBS-Tween) and incubated with samples diluted in PBS-Tween for 30 min. The detection system was the same as that used for aCL ELISA.

In-house aPS/PT ELISA was performed as previously described [21]. Briefly, medium binding microtiter plates (Corning Incorporated, Kennebunk, ME, USA) were coated with phosphatidylserine (Sigma-Aldrich, St. Louis, ZDA) in chloroform/methanol 1:4 and blocked with 1% bovine serum albumin (BSA, Sigma-Aldrich, St. Louis, ZDA) in Tris-buffered saline (TBS) containing 5 mM CaCl_2_ (1% BSA/TBS-Ca). Human prothrombin (Enzyme Research Laboratories, Ltd., Swansea, UK) [10 mg/L] and patient sera diluted 1:100 in 1% BSA/TBS-Ca were added to the wells immediately after each other and incubated for 1 h. After washing with 5 mM CaCl_2_-TBS-0.05% Tween 20, alkaline phosphatase-conjugated goat anti-human IgG/IgM (ACSC, Westbury, NY, USA) were applied in 1% BSA/TBS-Ca and incubated for 30 min. The detection system was the same as for aCL ELISA.

Lupus anticoagulant activity (LA) was determined four weeks after cessation of anticoagulant therapy in those patients in whom long-term anticoagulant therapy was not required according to guidelines. The assay was performed in blood samples collected in tubes containing 0.109 M sodium citrate. Platelet poor plasma was obtained by centrifugation at 2400× *g* for 20 min at 4 °C. After filtration, aliquots were stored at −80 °C until use. Clotting tests were performed using the BCS Siemens coagulation analyser according to the previous guidelines of the International Society on Thrombosis and Haemostasis ISTH [22]. Simplified Dilute Russell’s Viper Venom Test (dRVVT) was performed using LA1 Screening reagent and LA2 Confirmatory reagent (Siemens) following the company instructions [23]. A dRVVT ratio (LA1 screening/LA2 confirmatory) above 1.2 was considered positive for LA activity. The activity of LA was quantified as follows: low positive (LA1/LA2 = 1.2–1.5), medium (LA1/LA2 = 1.5–2.0) and high positive (LA1/LA2 > 2.0).

### 2.4. Statistical Analysis

Statistical analyses were performed using the IBM SPSS Statistics 26.0 program and GraphPad Prism 5.03 and included descriptive analyses, arithmetic means, medians and percentile values. Results are presented in tables and charts. Categorical variables were presented as numbers and percentages, and continuous variables as medians or means. For continuous variables, the normality of the distribution was checked using the Kolmogorov–Smirnov test. The significance of baseline differences was determined using the chi-square test (χ^2^) or the independent samples *t*-test. A *p* value of less than 0.05 was considered statistically significant for all statistical analysis.

## 3. Results

### 3.1. Patient Demographics and Risk Factors Analysis

Out of 278 consecutive DVT patients, 221 fulfilled the inclusion criteria and had at least five documented visits. There were 124 males and 97 females included in the study. The median age was 54 (range 18–86) years), while the average age was 52.5 years. Ultimately, 24/221 (10.9%) patients fulfilled APS classification criteria 24 weeks after DVT (13 male, 11 female, median age 58 (range 19–78 years)). The normality of the distribution for age was checked with the Kolmogorov–Smirnov test, showing a normally distributed variable (*p* = 0.055). There were no significant differences between the APS and non-APS groups as far as age (*t* = −0.05, *p* = 0.96) and sex (χ^2^ =0.04, *p* = 0.83) (Table 1) are concerned. For the majority of included patients, data on additional external DVT risk factors were available (Table 1) and there were no significant differences between the APS and non-APS groups.

### 3.2. Serological Characteristics of APS Patients

**Figure 1:**
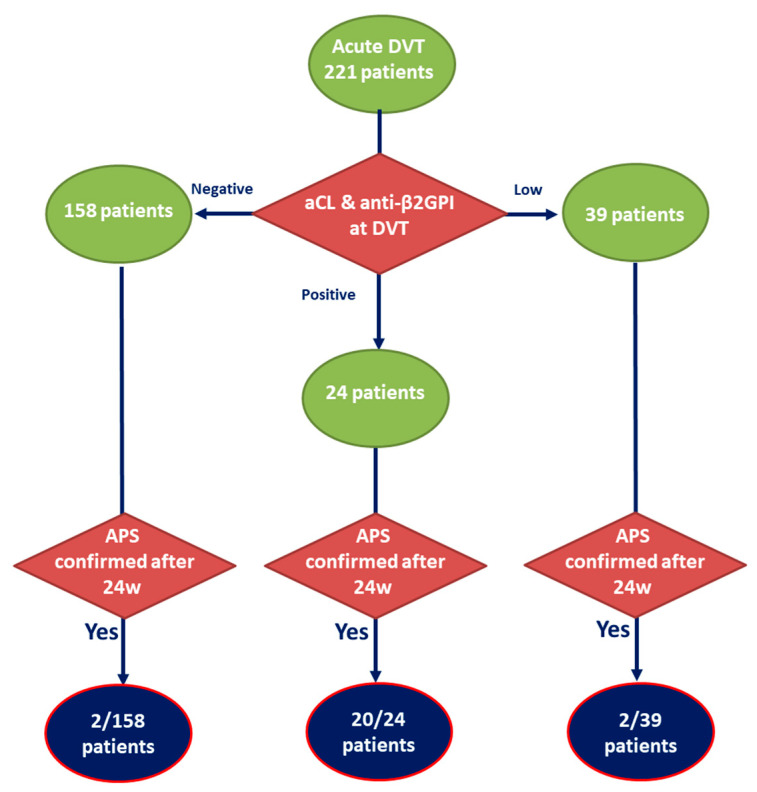
Schematic representation of participants’ aPL positivity according to diagnostic importance at the time of deep venous thrombosis (DVT) and number of patients who fulfilled current APS classification criteria 24 weeks (w) after DVT in each group. Negative: below detection limit; low: criteria aPL present between 95th and 99th percentile of healthy control population; positive: criteria aPL above 99th percentile of healthy control population.

Among the 24 APS patients, 20/24 (83.3%) met the criteria for aPL above the 99th percentile at the time of acute DVT. One patient (4%) had aCL IgG and one (4%) had aCL IgM between the 95th and 99th percentile of healthy blood donors. Two patients (8%) were negative for aCL and aβ2GPI, but later fulfilled APS criteria due to their positivitiy for LA at discontinuation of anticoagulant therapy (Figure 1).

**Figure 2:**
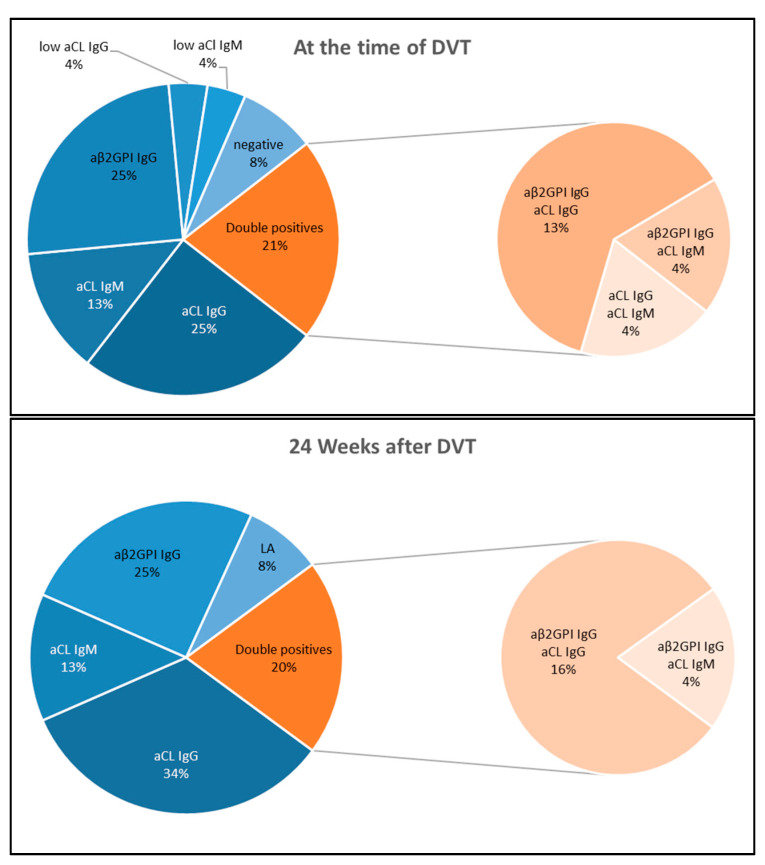
aPL profile of APS patients (*n* = 24) at the time of acute DVT (upper panel) and 24 weeks after DVT (lower panel).

The majority of APS patients (17/24, 71%) were single positive for aCL IgG, anti-β2GPI IgG or aCL IgM (Figure 2). Four patients (17%) were double positive for aCL IgG and anti-β2GPI IgG, one patient (4%) was double positive for aCL IgM and anti-β2GPI IgG, two patients (8%) had LA. One double positive patient, additionally, had anti-β2GPI IgM and low level aCL IgM; three double positive patients, additionally, had non criteria anti-β2GPI IgA present. One of the LA positive APS patients had persistently positive non criteria aPS/PT antibodies as well.

#### 3.2.1. Analysis of aPL Positivity at the Time of DVT

Out of 221 patients with DVT, 28 had positive values of either aCL, anti-β2GPI or aPS/PT at the time of the event. Twenty-four had criteria aPL, while four patients were single positive for non-criteria aPL, i.e., one for aPS/PT IgM and three for anti-β2GPI IgA. In 4/24 (16.7%) patients with aPL at the event, the levels of these aPL decreased in the following visits and were later identified as non-APS. Importantly, all of these patients were single aPL positive. The positive predictive value (PPV) of criteria aPL for APS at the time of the DVT was 84.0%.

158/221 patients with DVT had negative values of aPL at the time of the event. Out of these, two patients were diagnosed with APS due to their positivity for LA at discontinuation of anticoagulation therapy. Interestingly, one of the two LA positive patients had positive aPS/PT at the time of the event which also remained persistently positive during our follow-up.

39/221 patients with DVT had low positive values of aCL at the time of DVT. In two of these patients, APS was later confirmed due to their elevation of aCL levels at the subsequent visits.

The negative predictive value (NPV) of aPL values determined at the time of DVT (including aCL IgG/IgM and anti-β2GPI IgG/IgM) was 98.0%. Furthermore, the NPV of aPL, counting also aPS/PT IgG/IgM/IgA measurements at the time of the event, increased to 98.4%. The non-APS patient group had significantly lower levels of aCL and anti-β2GPI at the time of the event, and their levels continued to decrease over time, while aPL levels remained high in the APS group (*p* < 0.001).

##### Patients Positive for aCL IgG

**Figure 3:**
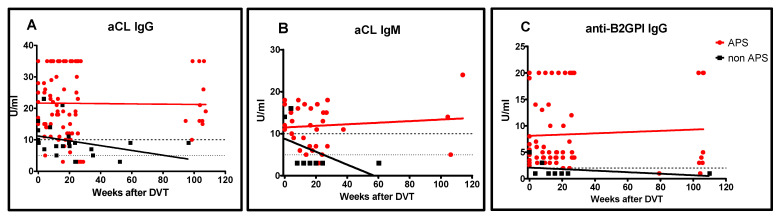
Prospective measurement of aCL IgG (**A**), aCL IgM (**B**) and anti-β2GPI (**C**) at the time of acute DVT and at repeated measurements over the following 120 weeks. The dark dotted lines represent the 99th percentile dividing the high positive values, and the light dotted line represents the 95th percentile dividing the low positive values.

Thirteen patients had positive aCL IgG levels at the time of the event. Eleven of these patients were later identified as having APS, while two did not, as their aCL IgG levels decreased at subsequent visits and did not reappear throughout the two-year follow-up (Figure 3). The PPV of aCL IgG for APS at the time of the event was 84.6% and the negative predictive value of aCL IgG was 93.8%.

##### Patients Positive for aCL IgM

Five patients had positive aCL IgM levels at the time of the event. Four of these patients were later identified as APS, while one was not, as his aCL IgM levels decreased at subsequent visits and remained negative throughout follow-up (Figure 3). The PPV of aCL IgM for APS at the time of the event was 80.0% and NPV 90.7%.

##### Patients Positive for Anti-β2GPI IgG

Thirteen patients had positive anti-β2GPI IgG at the time of the event and twelve of them were later diagnosed with APS. In one patient, the anti-β2GPI IgG levels decreased at subsequent visits and remained negative throughout follow-up (Figure 3). The PPV of anti-β2GPI IgG for APS at the time of the event was 92.9% and the NPV was 94.7%.

##### Patients Positive for Non-Criteria aPL

At the time of the event, five patients had positive aPS/PT (three IgG/IgM, one IgG only and one IgM only), all of whom were diagnosed with APS. Four of these patients were positive for criteria aPL at the same time, while the one who only had aPS/PT IgM was positive for aPS/PT alone. For this patient, the elevated levels of aPS/PT IgM persisted throughout the 120-week follow-up period; he was also LA positive.

One patient, who had criteria aCL IgG, was also positive for aCL IgA. None of the patients were single aCL IgA positive.

Five patients were positive for anti-β2GPI IgA. Two were concomitantly positive for anti-β2GPI IgG and diagnosed with APS, while three were negative for other aPL. Two of these three later became negative for anti-β2GPI IgA while one had persistently positive anti-β2GPI IgA.

## 4. Discussion

To our knowledge, this is the first prospective study with a two-year follow-up in patients with first acute DVT (provoked or unprovoked) to investigate the feasibility of using the determination of the aPL at the time of the acute thrombotic event for APS prediction. Out of 221 DVT patients included, 10.9% met current APS classification criteria [3]. This result is consistent with previously reported findings in which APS was confirmed in 9% of patients with first DVT [15], albeit in a younger patient population than ours. The demographics and the presence of additional external risk factors of our APS and non-APS cohorts of sequentially included patients were comparable. Since 1–5% of people in the general population are aPL positive, current guidelines suggest that aPL status should be tested in patients considered at risk for APS, i.e., patients younger than 50 years, patients with unprovoked arterial or venous thrombosis, patients with thrombosis at an unusual site or patients with recurrent thrombotic events [24]. However, our APS patient population had a median age greater than 50 years, similar to our non-APS group. Furthermore, the evenly distributed external DVT risk factors in APS and non-APS patients suggest that discontinuation of anticoagulation due to a provoked thrombotic event would put undiagnosed APS patients at risk of thrombosis as early as three months after the primary event.

Our major finding is a high NPV for APS (98.0%) of negative criteria aPL (aCL and anti-β2GPI G/M) at the time of the thrombotic event. Four (16%) APS patients were negative for criteria aPL at the time of the thrombotic event; two of which were later identified as LA positive after discontinuation of anticoagulant therapy, while in two aCL levels increased at the subsequent visits. aPL antibodies can disappear post-thrombosis. Interestingly, one of the LA positive patients also had positive non-criteria aPS/PT IgM antibodies that were persistently positive from the acute thrombotic event. Adding aPS/PT to criteria aPL determination at the time of the thrombotic event improved the negative predictive value to 98.4%; this is in line with our previous findings that additional APS patients could be determined with aPS/PT measurement [5].

Decreases in aCL titres have been reported in SLE patients after a thrombotic event [25]. In a recently published study, Khawaja et al. reported that in SLE-APS patients, complete loss of aPL positivity post thrombosis occurred in up to 51% for aCL IgM and 20% for LA [26]. In contrast, in our cohort of non-SLE-APS patients, the aPL titres remained stable in most patients and increased in two during the two-year observation after the thrombotic event.

The second important finding of this study is that 20/24 APS patients (83.3%) had positive values of the aCL and/or aβ2GPI at the time of the acute thrombotic event. The aPL positivity at the time of the event had an important 84% PPV for APS, suggesting that the positive aPL at the time of the thrombotic event can help us to decide on the duration of anticoagulant treatment of DVT patients and also raise the awareness of the possibility of APS in these patients. Several aPLs can occur transiently in association with viral and bacterial infections and probably other unspecified factors [27]. We also found 7/197 non-APS patients who had positive aPL levels at the time of DVT but whose levels later decreased, confirming the importance of repeated aPL measurement as implied in the international consensus statement on classification criteria for definite APS [3].

Strengths of our study include a prospective design and a relatively large DVT cohort that has been consecutively screened for aPL (largest sample to date) and independent blinded assessments of DVT and aPL status by a single expert aPL laboratory with over 20 years of experience. In addition, to our knowledge, this is the largest prospective study to date to determine the significance of persistent aPL after DVT.

Our study had limitations. Subjects were not tested for LA immediately after DVT and before starting anticoagulation therapy. Data on other established cardiovascular risk factors for APS, such as smoking, thrombocytopenia, hyperlipidemia and hypertension, were also not systematically collected. Nonetheless, aPL were measured blindly at each visit, and although additional risk factors might better estimate individual risk for recurrence, they would have little effect on aPL levels, which were the focus of this study.

In conclusion, this study followed 221 patients with DVT for aPL levels for at least two years after the index event. We identified 24 APS patients, the majority of whom (83.3%) already had elevated aPL levels at the time of DVT, including aCL, anti-β2GPI, and aPS/PT, which remained positive throughout follow-up. Our data show that a negative aPL level at the time of acute DVT greatly reduces the likelihood of patients having APS, whereas on the other hand, a positive aPL level has a strong positive predictive value for APS. Our results suggest that early aPL testing at the time of acute thrombotic events very accurately identifies potential APS patients and could be considered in therapeutic decisions.

## Figures and Tables

**Table 1:** Demoraphic and risk factor analysis of included deep vein thrombosis (DVT) patients. Results are expressed as median (range) for age, and counts (%) for sex and risk factors. Independent samples *t*-test was used for age, χ^2^ test was used for other categories.

	All N = 221 (100%)	Non-APS N = 197 (89.1%)	APS N = 24 (10.9%)	χ^2^ (*p* Value)
Age	54 (18–86)	54 (18–86)	58 (19–78)	*t* = −0.05 (0.96)
Sex	Male 124 (56.1%)	Male 111 (56.3%)	Male 13 (54.2%)	0.04 (0.83)
Injury	44 (28%)	41 (30.1%)	3 (14.3%)	2.27 (0.19)
Surgical procedure	16 (1.2%)	15 (11%)	1 (4.8%)	0.78 (0.70)
Immobilization	27 (17.2%)	25 (18.4%)	2 (9.5%)	1.00 (0.53)
Oral contraceptives	27 (40.9%)	25 (44.6%)	2 (20%)	2.13 (0.18)
Febrile state	10 (6.4%)	7 (5.1%)	3 (14.3%)	2.55 (0.13)
Longer flights	2 (1.3%)	2 (1.5%)	0 (0%)	0.31 (1.00)
Chronic comorbidities	6 (3.8%)	5 (3.7%)	1 (4.8%)	0.06 (0.58)
Family DVT history	20 (12.7%)	17 (12.5%)	3 (14.3%)	0.05 (0.73)

## Data Availability

All raw anonymized data fully available upon reasonable request.
